# Novel Colorimetric and Light Scatter Methods to Identify and Manage Peritoneal Dialysis-Associated Peritonitis at the Point-of-Care

**DOI:** 10.1016/j.ekir.2023.12.021

**Published:** 2023-12-30

**Authors:** Nishal Govindji-Bhatt, Stephnie M. Kennedy, Michael G. Barker, Darren Kell, Duncan Henderson, Nicholas Goddard, Ana Yepes Garcia, Adam S. Milner, Tom Willett, Ryan Griffiths, Peter Foster, William Kilgallon, Rachel Cant, Christopher G. Knight, David Lewis, Richard Corbett, Habib Akbani, Graham Woodrow, Bhrigu Sood, Osasuyi Iyasere, Simon Davies, Junaid Qazi, Anand Vardhan, Laura Gillis, Martin Wilkie, Curtis B. Dobson

**Affiliations:** 1 Microbiosensor Ltd., The Incubator Building, Manchester, UK; 2 Division of Pharmacy and Optometry, School of Health Sciences, The University of Manchester, Manchester, UK; 3 Division of Cancer Sciences, School of Medical Sciences, The University of Manchester, Manchester, UK; 4 Department of Earth and Environmental Sciences, School of Natural Sciences, The University of Manchester, Manchester, UK; 5 Salford Royal NHS Foundation Trust, Salford, UK; 6 Imperial College Healthcare NHS Trust, Hammersmith Hospital, Imperial College Healthcare NHS Trust, London, UK; 7 Bradford Teaching Hospitals NHS Foundation Trust, St Luke’s Hospital, Bradford, UK; 8 Leeds Teaching Hospitals NHS Trust, St James’s University Hospital, Leeds, UK; 9 Epsom and St Helier Hospitals NHS Trust, Surrey, UK; 10 John Walls Renal Unit, University Hospitals of Leicester NHS Trust, Leicester, UK; 11 University Hospital of North Midlands NHS Trust, Stoke-on-Trent, UK; 12 Manchester University NHS Foundation Trust, Manchester UK; 13 Sheffield Teaching Hospitals NHS Foundation Trust, Sheffield, UK

**Keywords:** diagnosis, peritoneal dialysis, peritonitis, leukocyte

## Abstract

**Introduction:**

Peritoneal dialysis (PD)-related peritonitis (PDRP) is a common cause of transfer to hemodialysis, patient morbidity, and is a risk factor for mortality. Associated patient anxiety can deter selection of PD for renal replacement therapy. Diagnosis relies on hospital laboratory tests; however, this might be achieved earlier if such information was available at the point-of-care (POC), thereby significantly improving outcomes. The presence of culturable microbes and the concentration of leukocytes in effluent both aid peritonitis diagnosis, as specified in the International Society for Peritoneal Dialysis (ISPD) diagnostic guidelines. Here, we report the development of 2 new methods providing such information in simple POC tests.

**Methods:**

One approach uses a tetrazolium-based chemical reporting system, primarily focused on detecting bacterial contamination and associated vancomycin-sensitivity. The second approach uses a novel forward light-scatter device (QuickCheck) to provide an instant quantitative cell count directly from PD patient effluent.

**Results:**

The tetrazolium approach detected and correctly distinguished laboratory isolates, taking 10 hours to provide non-quantitative results. We compared the technical performance of the light scatter leukocyte counting approach with spectrophotometry, hemocytometer counting and flow cytometry (Sysmex) using patient effluent samples. QuickCheck had high accuracy (94%) and was the most precise (coefficient of variation <4%), showing minimal bias, overall performing similarly to flow cytometry.

**Conclusion:**

These complementary new approaches provide a simple means to obtain information to assist diagnosis at the POC. The first provides antibiotic sensitivity following 10 hours incubation, whereas the second optical approach (QuickCheck), provides instant accurate total leukocyte count.

## Graphical abstract

**Figure:**
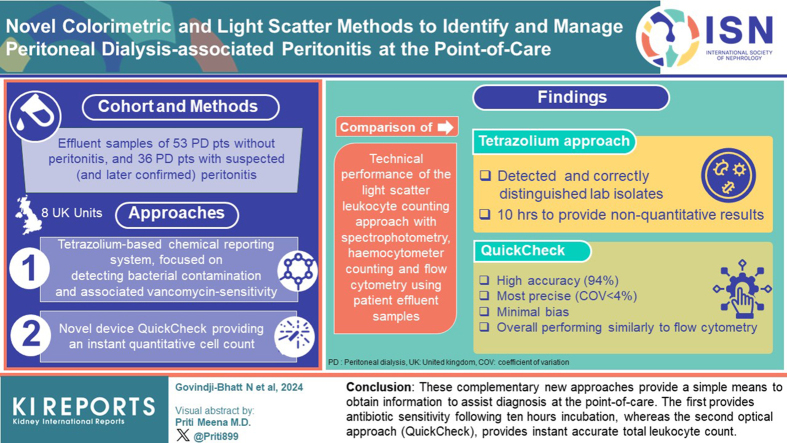

PDRP is a major cause of transfer of patients to hospital-based hemodialysis^1^, ^2^, ^3^, ^4^ with 2% to 6% associated mortality,^5^ shown to be associated with an elevated hazard ratio for PD discontinuation and death.^6^ The risk of PDRP is an ever-present concern for people who receive PD, due to difficulty identifying PDRP symptoms and infection prevention measures,^7^ reducing selection of PD.^8^ Peritonitis management involves symptom recognition and visual assessment of effluent cloudiness, travel to the clinic for assessment and hospital cytology, and finally antibiotic therapy. Development of cloudy effluent is an early indicator of PDRP and reflects presence of leukocytes.^9^ Its assessment is qualitative and subjective, resulting in false positives and false negatives. Some patients present without abdominal pain despite cloudy effluent, and vice versa.^10^ Prompt diagnosis relies heavily on the patient being able to visually examine the spent dialysate, which is clearly a challenge for those with visual impairment. Once PDRP is suspected, diagnosis requires 2 out of 3 indicators: >100 cells/μl leukocytes (with >50% polymorphonuclear leukocytes), microbial culture, and patient signs or symptoms.^10^

Following diagnosis, prompt antibiotic therapy is critical, with each hour of delay from initial presentation increasing risk of PD failure or death by 6.8%.^11^ Further delays may be introduced by transferring effluent samples for leukocyte assessment by central testing laboratories in the same hospital or at another location. PDRP outcomes may be improved by POC peritonitis detection, either by the healthcare professional in clinic, or as a screen by patients at home. Technical approaches to achieve this could include microbial detection, monitoring biochemical changes associated with leukocytes, or direct leukocyte quantification.

Given that effluent from patients with PDRP is frequently culture negative, this may limit the usefulness of microbial detection as a generic diagnostic. UK culture negative rates are reported at 5% to 38% depending on the center,^12^ suggesting potentially high false negative rates for a microbial-based PDRP POC diagnostic. Touch or environmental contamination with bacteria is also possible with this approach (unlike leukocyte detection) risking false positives. Moreover, recent polymerase chain reaction approaches to identify bacteria in patients with PDRP have proved unsuccessful.^13^ Nonetheless, POC microbial antibiotic sensitivity information may inform antibiotic therapy selection, at least in culture positive peritonitis cases, and so this might be considered as an additional component of a PDRP POC diagnostic.

Recently, a single-use lateral flow device using immune markers as a proxy for leukocytes has been approved. This detects matrix metalloproteinase-8 and interleukin 6. Matrix metalloproteinase-8 is associated with activated neutrophils, whereas effluent interleukin 6 effluent concentrations increased during early PRRP.^14^ A positive test prompts full clinical assessment and leukocyte assessment, which once complete, allows diagnosis under ISPD criteria. As such, this test replaces the initial assessment of cloudiness with a less subjective screen, though it does not expedite obtaining a leukocyte count.

In the present study, we examined the status of leukocytes in patients without peritonitis to confirm the 100 cells/μl threshold, and characterize those leukocytes present in patients without peritonitis. We then developed 2 different methods to provide POC information to assist in PDRP diagnosis. The first method uses a chemical test based on tetrazolium indicators (widely used in research to quantify cell viability). Tetrazolium compounds were tested to identify those developing intensely-colored formazans after incubation with microbial and eukaryotic cells, clearly visible to the eye.^15^ Various inhibitors were tested to enable selective assessment of leucocytes, vancomycin-sensitive bacteria, and vancomycin-insensitive bacteria. Three chemical formulations were defined to indicate either the presence of vancomycin-sensitive or insensitive microbes (formulation 1 and 2), or the presence of >100 leukocytes/μl (in formulation 3), with the test performed in a bespoke cassette and incubator. The second approach uses a novel forward light scattering device (QuickCheck), which provides an instant leukocyte count directly from a small sample of dialysis effluent. We compared its accuracy, precision, and bias with that of absorbance measurements (another optical method) and with flow cytometry (Sysmex) or hemocytometer counting (standard UK hospital cytology methods).

## Methods

### PD Effluent Samples

Effluent samples were collected from 53 PD patients without peritonitis, and 36 PD patients with suspected (and later confirmed) peritonitis from 8 UK renal units (Salford Royal NHS Foundation Trust, Imperial College Healthcare NHS Trust, Bradford Teaching Hospitals NHS Foundation Trust, Leeds Teaching Hospitals NHS Trust, Epsom and St Helier Hospitals NHS Trust, University Hospitals of Leicester NHS Trust, Manchester University NHS Foundation Trust, and Sheffield Teaching Hospitals NHS Foundation Trust). Samples were processed within 4 hours. Study protocols were reviewed and approved by NW Greater Manchester West Research Ethics committee (18/NW/0765/IRAS ID 250220). Total leukocyte counts were determined by each center, using standard hospital hemocytometer or Sysmex counting.

Leukocyte and bacterial counts in PD effluent were also performed independently at a single laboratory for further PD patient samples (30 with and 96 without peritonitis). Study protocols were approved by HRA and Health and Care Research Wales (HCRW) (21/WM/0033/IRAS ID 294250). Cell counts were carried out using a Sysmex UF5000, Body Fluid function mode, and calibrated with Sysmex UF control beads (Sysmex, Milton Keynes, UK) and 600 μl samples were analyzed**.** This flow cytometric instrument is commonly used in hospital laboratories to enumerate leukocytes and bacteria in multiple clinical samples types, using a light scatter approach, and is approved for used with PD effluent.

### Mass Cytometry

Sample processing is described in the Supplementary Methods, along with the staining panel (Supplementary Table S1). Analysis was performed using FlowJo; normalization beads (used in sample acquisition) were removed from the gating, and dead cells removed by gating on the cisplatin negative population. Single cell population was gated based on 191Ir /193Ir expression, and single cell live population exported to the R package Cytofkit. Identification of immune cell population was determined through clustering analysis, based on expression of cell surface markers, and percentage of immune cell subsets calculated.

### Bacterial Strains and Cell Lines

*Staphylococcus aureus* (ATCC 6538P, ATCC 25923, ATCC 29213, ATCC 43300), *S* *epidermidis* (ATCC 12228, ATCC 14990, ATCC 35983), *Acinetobacter baumanii* (ATCC 19606), *Pseudomonas aeruginosa* (ATCC 9027, ATCC 23997*,* ATCC 27853), *Klebsiella pneumoniae* (ATCC 8044, ATCC 700603), *Escherichia coli* (ATCC 8739, ATCC 10536), *Serratia marcescens* (ATCC 8100), *and Enterococcus faecalis* (ATCC 51299) were obtained from ATCC (Virginia). All were grown at 37 °C in tryptone soy broth (Oxoid, Ltd, Hampshire, UK) with agitation at 200 rpm, with growth estimated by absorbance at 600 nm (Biochrom Ltd. Cambridge, UK). Bacterial concentrations were routinely verified on tryptone soy agar (TSA) (Oxoid Ltd, Hampshire, UK).^16^ HL-60 cells (ECACC 98070106, Public Health England, Salisbury, UK) were grown in Iscove’s Modified Dulbecco’s Medium (Gibco) supplemented with 20% heat inactivated fetal bovine serum (Sigma-Aldrich, Dorset, UK) and 4 mM L-glutamine (Sigma-Aldrich). Jurkat cells (Jurkat E6.1, ECACC, Public Health England, Salisbury, UK) were cultured in Roswell Park Memorial Institute-1640 (RPMI-1640) media (Gibco, Paisley, UK), containing 2 mM L-glutamine (Sigma-Aldrich), supplemented with 10% heat inactivated fetal bovine serum. Mammalian cells were incubated at 37 °C and 5% CO_2_ in a humidified incubator.

### Measuring Thresholds for Visible Color Change

Indicator formulations (see Supplementary Materials for experiments to define their components) were incubated with 16 separate laboratory bacteria (0.1–1 × 10^4^ CFU/μl) or HL60 cells (300–1 × 10^4^ cells/μl). Threshold to produce a visible color change was determined after 10 hours incubation at 37 °C. Details for observing color change in full size cassettes are presented in the Supplementary Methods.

### Effect of Formulation Encapsulation

The final indicator formulations (Supplementary Table S2) were prepared as loose powder and in pharmaceutical capsules and were added to 16 ml of filtered PD effluent, and placed in polyvinyl chloride (PVC) chambers. Formulations 1 and 2 were both inoculated with 100 CFU/μl *S aureus* (ATCC 6538P) or *P aeruginosa* (ATCC 9027) or an uninoculated control. Formulation 3 was treated with HL60 cells at 500 cells/μl and uninoculated control. These were incubated for 10 hours at 37 °C, before visual assessment of color development.

### Light Scatter Leukocyte Counter

The instant cytometer was constructed to allow forward light scatter measurements from PD effluent samples (this principle has previously been used for cell counting).^17^ The device consisted of a 5 mW 655 nm collimated laser diode light source, which passes through a sample of effluent (1 cm light path). Twelve photodiode sensors quantify scattered light up to 12° from the incident axis, enabling the intensity of forward light scattered by interactions with cells in suspension to be characterized. The laser is mounted on a printed circuit board, with a second printed circuit board housing photodiodes. After we established the relationship between light scatter and leukocyte concentration, software was developed for calculating leukocyte count based on scattering measurements. Cell count is displayed on a liquid crystal display screen.

### Accuracy, Precision, and Bias for 4 Cell Counting Methods

Leukocytes were isolated from PD effluent samples by centrifugation at 400*g* for 5 minutes. Supernatants were discarded and any red blood cells removed using 5 ml dH_2_O (30 s), followed by neutralization with 5 ml of 1.8% NaCl (Sigma-Aldrich). Jurkat cells and clinically-derived leukocytes were both subjected to centrifugation at 400*g* for 5 minutes and cell pellets resuspended in 5 ml of 0.22 μm filtered effluent, counted using flow cytometry (Sysmex UF-5000) (Sysmex UK Ltd, Milton Keynes, UK), and diluted in filtered effluent to 1 × 10^4^ cells/μl. Test cell concentrations were prepared by serial dilution in effluent to produce 10, 25, 50, 75, 100, 500, and 1 × 10^3^ cells/μl.^18^ For each cell counting method, measurements were taken simultaneously and performed blind by independent operators in a randomized order. Further details are provided in the Supplementary Methods.

### Correlation of Cell Counts in Patient Effluent Between QuickCheck and Sysmex Devices

Effluent samples of unknown cell concentrations, from randomly selected patients were collected from 13 patients without peritonitis, 9 patients with suspected peritonitis, and 7 patients with peritonitis on antibiotic therapy within 4 hours of sample drain. Samples were received on separate days and measured independently. Patient effluent was added to cuvettes and measured simultaneously on the QuickCheck device, and using a Sysmex UF-5000, as previously. To prevent bias, cell counts on each cell counting device were performed blind by independent operators. Each sample was measured in triplicate and average data shown. Linearity of the data was determined for QuickCheck and Sysmex UF-5000.

### Potential Impact of Bacteria on QuickCheck Counts

Various concentrations of *E coli* (ATCC 10536) and *S* *aureus* (ATCC 6538P) were prepared (1–1 × 10^4^ CFU/μl) in filtered patient effluent, and each used as a test sample in a QuickCheck device. The resulting apparent ‘leukocyte count’ reported by the instrument was recorded.

### Statistical Analysis

Statistical analysis was performed using GraphPad Prism (GraphPad Software, CA). Normality of data was tested (with Shapiro–Wilk) before performing nonparametric tests (*P* > 0.05). Mann Whitney test was used to compare 2 conditions; and for multiple comparison tests, we used a 1-way analysis of variance with a Kruskal Wallis test. Agreement between methods was tested using linear regression analysis, Pearson, and intraclass correlation coefficients (R)^19^ (Supplementary References). Bland-Altman analysis for bias was performed with 95% confidence (±2 SD) intervals calculated on Log_10_ transformed data. *P <* 0.05 was considered significant. Further details are provided in Supplementary Methods.

## Results

### Leukocytes and Bacteria in PD Effluent

**Figure 1:**
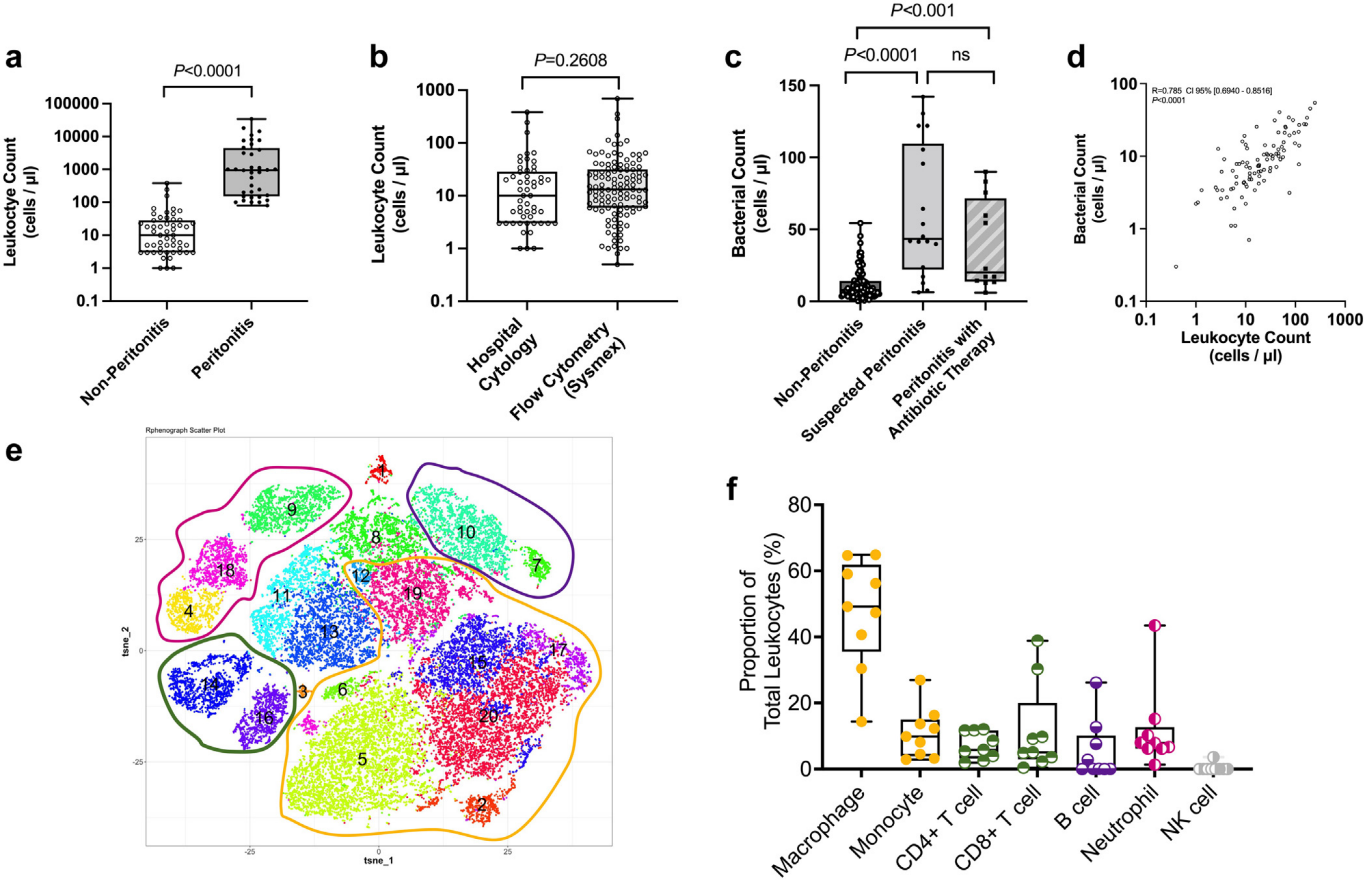
Cytological analysis of effluent from PD patients with or without peritonitis. (a) Total leukocyte counts in effluent samples from 53 PD patients without peritonitis or 36 PD patients with suspected (later confirmed) peritonitis determined by standard hospital cytology in eight UK clinics, presented as a box plot. *P* < 0.0001 using a Mann-Whitney (two-tailed) comparison test (b) Comparison of total leukocyte counts for effluent from 53 non-peritonitis patients from hospital cytology in eight UK clinics with those for 118 non-peritonitis PD patients, using flow cytometric analysis (Sysmex) in a single laboratory, presented as a box plot. *P* = 2.608 using a Mann-Whitney (two-tailed) comparison test (c) Total bacterial counts in effluent samples from 96 PD patients without peritonitis, 18 PD patients with suspected peritonitis and 12 PD patients with peritonitis and antibiotic therapy determined by flow cytometry (Sysmex), presented as a box plot. Statistical analysis performed using a Kruskal-Wallis one-way ANOVA, post-hoc Dunn’s multiple comparison test. (d) Bacterial counts from (c) plotted against total leukocyte counts for patients without peritonitis. Correlation shown using Pearson's correlation co-efficient (R = 0.7852) and 95% confidence intervals [0.6940, 0.8516]. *P* < 0.0001 (two-tailed, alpha 0.05). (e) and (f) Leukocyte sub-populations in effluent from PD patients without peritonitis. Cells from peritoneal dialysis fluid of 9 non-peritonitis patients were subjected to mass cytometry, using an antibody panel for immune markers. Sub-populations were identified based on their immune marker expression. Heat maps were generated to identify different immune cell populations, including macrophages and monocytes (clusters 2, 5, 6, 12, 15, 17, 19 and 20), T cells (clusters 14 and 16), B cells (clusters 7 and 10) and neutrophils (clusters 4, 9 and 18). Box plot shows the proportion of total leukocytes as percentages.

Average hospital leukocyte counts in PD effluent from patients without peritonitis (*n* = 53) was (30.2, SD: ±63.6) + (range 1–382 cells/μl), as expected, significantly lower than counts in patients with peritonitis (3686, SD: ±6762 (range 80–34020 cells/μl), *n* = 36; *P* < 0.001). Three patients without peritonitis had leukocyte counts above 100 cells/μl (the ISPD specified threshold). Of the remainder, 27 (51%) were in the range 11 to 100 cells/μl and 25 (47%) had <10 cells/μl. Only 1 of the 36 patients with peritonitis (2.7%) had a below threshold hospital cytology count (80 cells/μl; diagnosis confirmed by positive microbiological culture), with 21 (58%) having counts in the range 100 to 1 × 10^3^ cells/μl, 10 (27%) in the range 1.001 × 10^3^ to 1 × 10^4^ cells/μl, and 4 (11%) > ×10^4^ cells/μl (Figure 1a). We found that the lowest level that cloudiness begins to be detectible visibly is 500 cells/μl (Supplementary Figure S1), and so cloudiness may not be clearly visible for the 39% of peritonitis patients with counts of 100 to 500 cells/μl.

We compared counts for nonperitonitis samples from all 8 hospitals laboratories with those for a non-peritonitis cohort counted using a single Sysmex flow cytometer. Multiple hospital counts (*n* = 53) were 30.2 (SD: ±63.6) and single flow cytometer counts (*n* = 118) were (33.4, SD: ±76.8), showing no significant difference, confirming counting consistency for the multiple sites (*P* = 0.26) (Figure 1b).

We next measured bacteria and leukocytes in effluent from a cohort of PD patients without peritonitis (*n* = 96), and with peritonitis, either before (*n* = 18) or during antibiotic therapy (*n* = 12). Sysmex mean bacterial count in non-peritonitis samples was 11.4 (SD: ±10.3) bacterial cells/μl; for peritonitis before antibiotics, it was 61.9 (SD: ±45.7) cells/μl; and with antibiotics, it was 38.9 (SD ±31.4) cells/μl (Figure 1c). Bacterial levels were significantly higher in patients with peritonitis with or without antibiotics compared with non-peritonitis patients (*P* = 0.0001 and *P* = 0.0005, respectively). Because bacteria were consistently detected in supposedly “sterile” effluent from patients without peritonitis, we examined relative levels of leukocytes in these samples to rule out bacterial levels simply reflecting background contamination during sampling. There was a strong correlation between leukocyte and bacterial concentration overall (R = 0.785) (Figure 1d), suggesting that bacteria were genuinely present within the peritoneal cavity, due to the corresponding leukocytic response.

Mass cytometry revealed many leukocyte subtypes in PD patients without peritonitis, with macrophages being most common (47%, SD: ±16.8%). Other types were detected though these appeared more variable, and included monocytes (11%, SD: ±7.7%), CD4+ T-Cells (7% , SD: ±4.1%), CB8+ T-Cells (12%, SD: ±13.5%), B-Cells (6%, SD: ±8.9%), neutrophils (12%, SD: ±12.4%), and NK cells (0.4%, SD: ±1.2%) (Figure 1e and f).

### Tetrazolium Indicator Selection

We tested color change of different tetrazolium compounds after exposure to gram-negative, gram-positive, and mammalian cells. WST-9 with PVP/PMS (WST-9-PP) underwent a strong visible color change on exposure to all concentrations of bacteria (Supplementary Figure S2A), even those at the low levels detected in effluent from patients with PD (<1.4 × 10^3^ CFU/ml). Three tetrazolium compounds (MTT, TV, and BTC) did not undergo a color change for most concentrations of *S aureus*, suggesting toxicity for these against this gram-positive organism. WST-9-PP also produced the strongest color with HL60 cells, with color visible at >100 cells/ml (Supplementary Figure S2A).

### Chemical POC Method: Selection Factors

Incubation of high mammalian cell levels (1 × 10^4^ cells/ml) with 0.005% digitonin prevented any color change, and so this was included in microbe-sensitive formulations 1 and 2; this concentration did not inhibit reduction of WST-9-PP by 3 gram-positive or 3 gram-negative organisms (Supplementary Figure S2B). Selective inhibition was confirmed using mixtures of bacteria and HL60 cells. No color change was apparent for HL60 cells alone (up to 1 × 10^4^ cells/ml), but a strong color change was visible for any well containing >1 CFU/ml *S* *aureus* or *E* *coli*, the lowest bacterial level in PD effluent (Supplementary Figure S2C). The ability of up to 3 antibiotics to suppress growth of diverse microbes was tested as the basis for the mammalian cell selective formulation 3. Only media containing all 3 completely inhibited growth, though that did not affect WST-9-PP reduction by HL60 cells (Supplementary Figure S2D). No color change was apparent for bacteria alone (we tested up to 1 × 10^4^ CFU/ml), or with HL60 cells up to 100/ml, but a strong color change occurred for those wells containing HL60 cells at >300/ml (Supplementary Figure S2E).

We found that 16μg/ml vancomycin was sufficient to discriminate vancomycin-sensitive from vancomycin-insensitive strains (Supplementary Table S3), and this was then tested for higher volume samples enclosed in transparent PVC reaction chambers. After 10 hours incubation, formulations 1 and 2 showed an identical intense blue-purple color change for the 2 gram-negative organisms, but for the 2 gram-positive organisms this occurred with formulation 1 only (Supplementary Figure S2F).

The final 3 formulations were challenged with 16 bacteria, or HL60 cells, at various concentrations. All bacteria caused color development in formulation 1, with 15 of 16 producing this at 100 CFU/ml or higher. Formulation 2 changed color for all 8 gram-negative (vancomycin-insensitive) strains at the same threshold, but did not detect the gram-positive organisms (even at 1 × 10^4^ CFU/ml). None of the 16 bacterial strains caused any color change in formulation 3 (mammalian cell indicator) but color was produced for HL60 cells at >300 cells/ml (Table 1).

**Table 1:** Appearance of 3 formulations after 10-hour incubation with vancomycin-sensitive and vancomycin-insensitive bacteria or HL60 cells a

Bacterial strain and ATCC reference	Formulation 1 (all bacteria)						Formulation 2 (vancomycin-insensitive bacteria)						Formulation 3 (mammalian cells)					
	10^4^	10^3^	100	10	1	0.1	10^4^	10^3^	100	10	1	0.1	10^4^	10^3^	100	10	1	0.1
*S epidermidis* 14990	+	+	+	-	-	-	-	-	-	-	-	-	-	-	-	-	-	-
*S epidermidis* 35984	+	+	+	-	-	-	-	-	-	-	-	-	-	-	-	-	-	-
*S epidermidis* 12228	+	+	+	+	+	-	-	-	-	-	-	-	-	-	-	-	-	-
*S epidermidis* 35983	+	+	+	+	-	-	-	-	-	-	-	-	-	-	-	-	-	-
*S aureus* 43300	+	+	+	+	+	+	-	-	-	-	-	-	-	-	-	-	-	-
*S aureus 25923*	+	+	+	-	-	-	-	-	-	-	-	-	-	-	-	-	-	-
*S aureus 29213*	+	+	+	+	+	-	-	-	-	-	-	-	-	-	-	-	-	-
*S aureus 6538*	+	+	+	-	-	-	-	-	-	-	-	-	-	-	-	-	-	-
*A baumannii* 19606	+	+	+	-	-	-	+	+	+	-	-	-	-	-	-	-	-	-
*P aeruginosa 27853*	+	+	+	-	-	-	+	+	+	-	-	-	-	-	-	-	-	-
*P aeruginosa 9027*	+	+	+	-	-	-	+	+	+	-	-	-	-	-	-	-	-	-
*P aeruginosa 23997*	+	+	-	-	-	-	+	+		-	-	-	-	-	-	-	-	-
*K pneumoniae 8044*	+	+	+	+	+	-	+	+	+	+	+		-	-	-	-	-	-
*K pneumoniae 700603*	+	+	+	+	+	-	+	+	+	+	+		-	-	-	-	-	-
*E coli* 8379	+	+	+	+	+	+	+	+	+	+	+	+	-	-	-	-	-	-
*Sa marcescens* 8100	+	+	+	-	-	-	+	+	+	-	-	-	-	-	-	-	-	-

	10^4^	10^3^	100	10	1	0.1	10^4^	10^3^	100	10	1	0.1	10^4^	10^3^	500	300	100	10
HL60 cells	-	-	-	-	-	-	-	-	-	-	-	-	+	+	+	+	-	-

*A baumannii*, *Acinetobacter baumannii; E coli, Escherichia coli; K pneumoniae*, *Klebsiella pneumoniae; P aeruginosa*, *Pseudomonas aeruginosa; S aureus*, *Staphylococcus aureus; S epidermidis*, *Staphylococcus epidermidis; Se marcescens*, *Serratia marcescens*.

a + indicates visible purple colour change; − indicates no discernible colour change. Values are CFU/ml or cells/ml.

### POC Cassette

**Figure 2:**
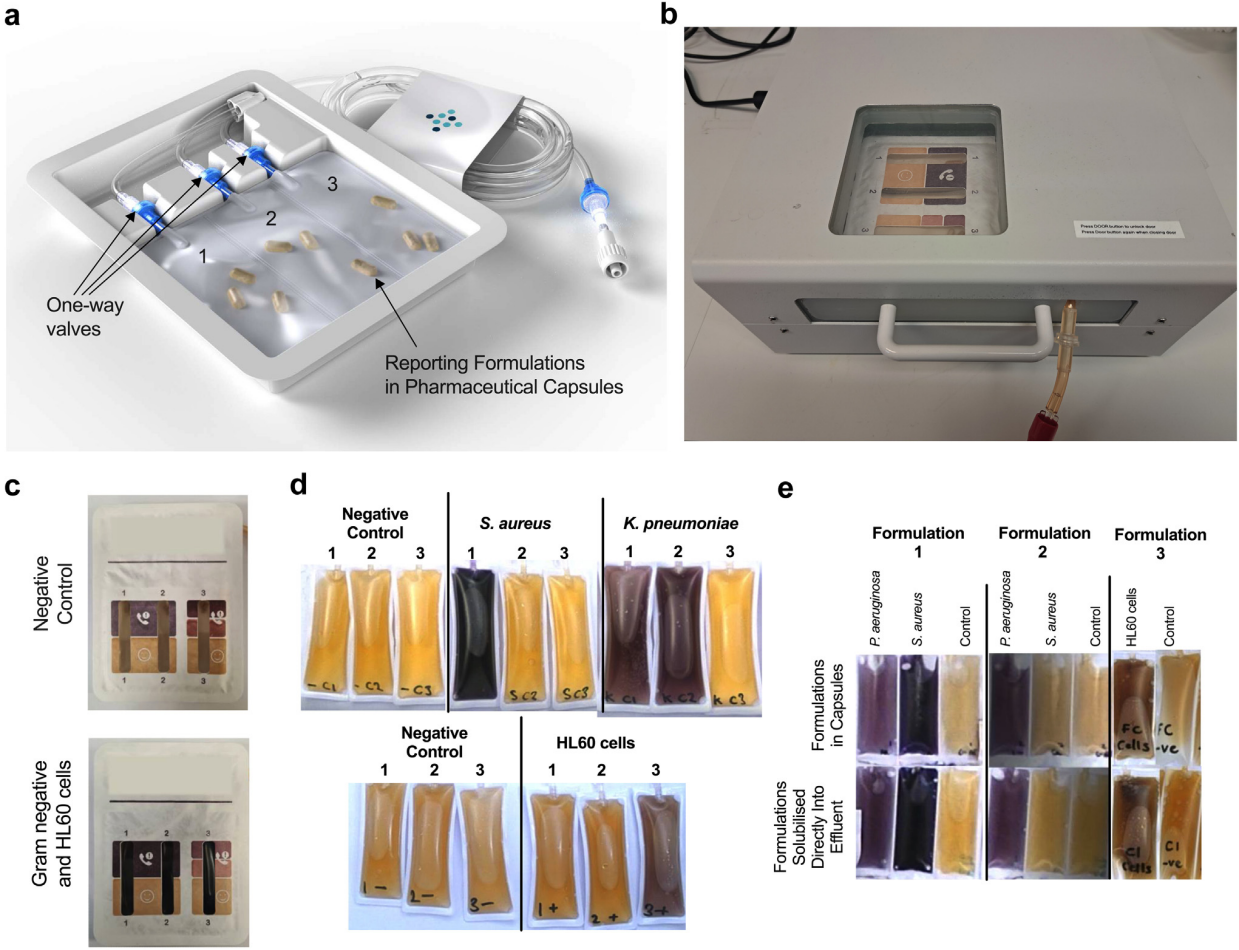
Point-of care cassette to incubate PD effluent with the three colorimetric formulations. (a) Disposable cassette comprising three connected PVC chambers, with each chamber containing soluble pharmaceutical capsules, containing the three chemical formulations. Multiple capsules are used for each formulation to maximise shelf-life (avoiding incompatible chemicals being stored together). One-way values prevent back-flow of effluent and potential leakage of contaminated fluid and chemicals. (b) Prototype 37 °C incubator, suitable for use in clinic. Cassettes are placed in a cradle prior to sliding this into incubator, and beginning 10h heating cycle. (c) After incubation completed, each window was viewed to compare colour of test formulation with the positive and negative colours printed on the upper surface of the cassette. Negative control cassette treated with sterile effluent, and positive control treated with 1x10^4^ CFU/μl of a gram-negative organism (*Ecoli* 10536) and 3× 10^3^ cells/μl Jurkat cells. (d) Visual appearance of three chambers of cassette after treatment with PD effluent containing 1× 10^2^ CFU/μl *Saureus* (vancomycin-sensitive) or *Kpneumoniae* (vancomycin-insensitive), or 5 × 10^2^ cells/μl HL60 cells. Colour change in formulation 1 but not 2 indicates presence of vancomycin-sensitive (gram-positive) organisms, colour change in both formulations 1 and 2 indicates vancomycin-insensitive (gram-negative) organisms, whereas colour change in formulation 3 indicates >300 leukocytes/µl. (e) Comparison of colour change observed in cassette, after challenge of each formulation either solubilised directly as powder in effluent before adding to PVC chambers, or solubilised in capsule form. Images in panels (c), (d), and (e) are representative of typical results (all experiments were carried out in triplicate). The appearance of images have not been altered or modified from their original format at the time of capture.

Test cassettes were constructed, fillable by syringe, with powdered formulations placed in soluble pharmaceutical capsules within each PVC chamber (Figure 2a). After a 10-hour incubation at 37 °C (Figure 2b), the color in each chamber can be viewed through the transparent window and compared with printed colors on the cassette lid (Figure 2c). Test samples of effluent containing *S* *aureus* (gram-positive), *K* *pneumoniae* (gram-negative) and HL60 cells caused color change in the expected formulations (Figure 2d). Color intensity and time to develop was identical for formulations solubilized directly into effluent or provided dry enclosed in capsules (Figure 2e).

### Forward Light Scatter Device: Development and Testing

**Figure 3:**
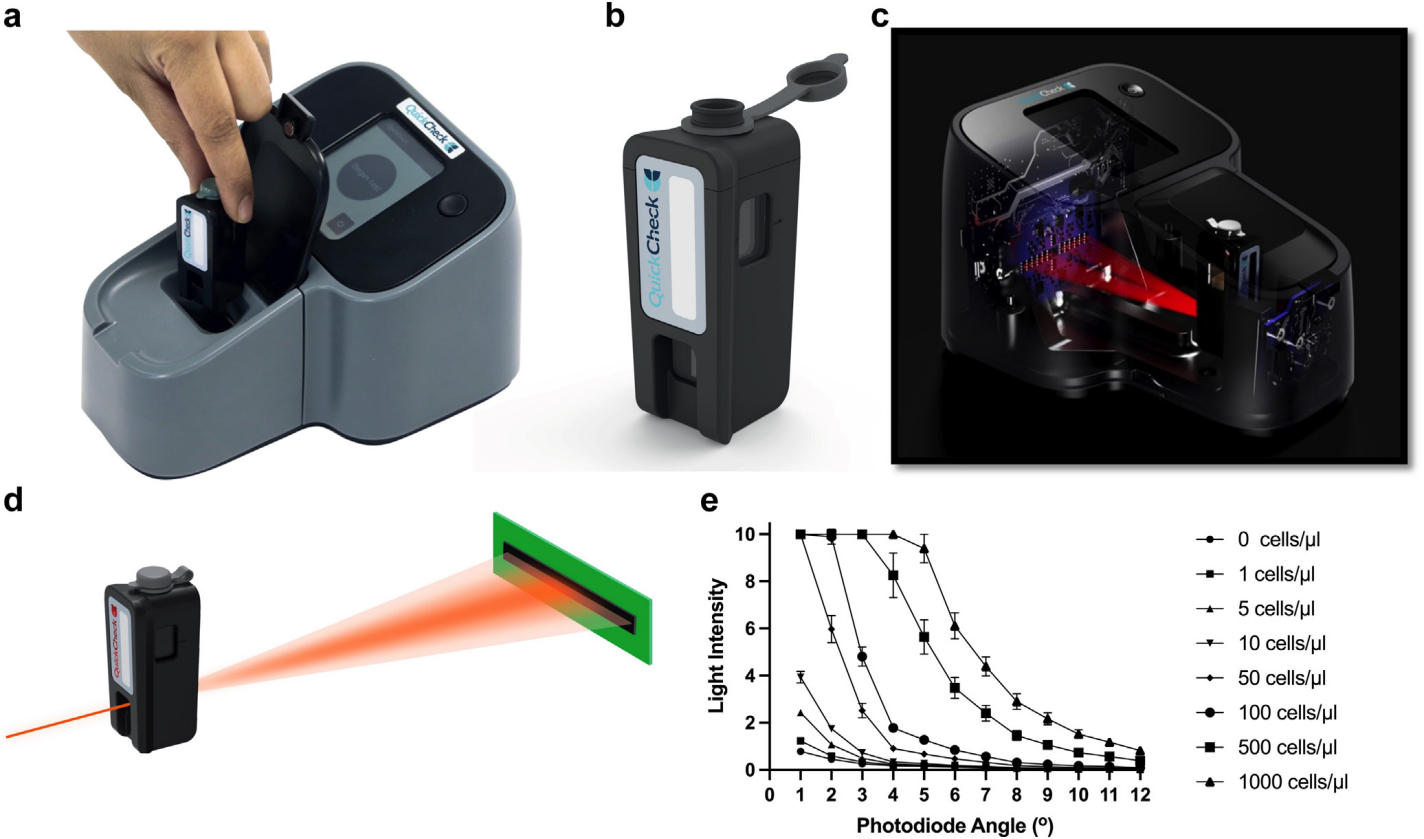
Forward laser light scatter device for instant enumeration of leukocytes in PD effluent. (a) A prototype forward light scatter detection instrument was constructed using commercially available laser light source and photodiodes positioned at different angles from the incident light source beam. (b) Sample containers were 3-D printed, able to hold up to 2.5 ml PD effluent, with two optically clear windows, one permitting entry of laser light, and the allowing scattered light emerging on the opposite side of the container to continue unobstructed to the photo-detectors. (c) Overall instrument design, showing laser light illuminating a sample within the instrument, and scattered light detected up to 12° from the axis of the incident light source. (d) Path of laser light entering the sample container, and undergoing forward scatter on encountering leukocytes suspended in effluent. (e) Light intensity at 12 photodiodes positioned at different angles within the instrument, after introducing various concentrations of Jurkat cells, suspended in filtered PD effluent, into the light path. Error bars shown are ±SD, and data are from triplicate measurements of a single replicate in three independent experiments (*n* = 3).

**Figure 4:**
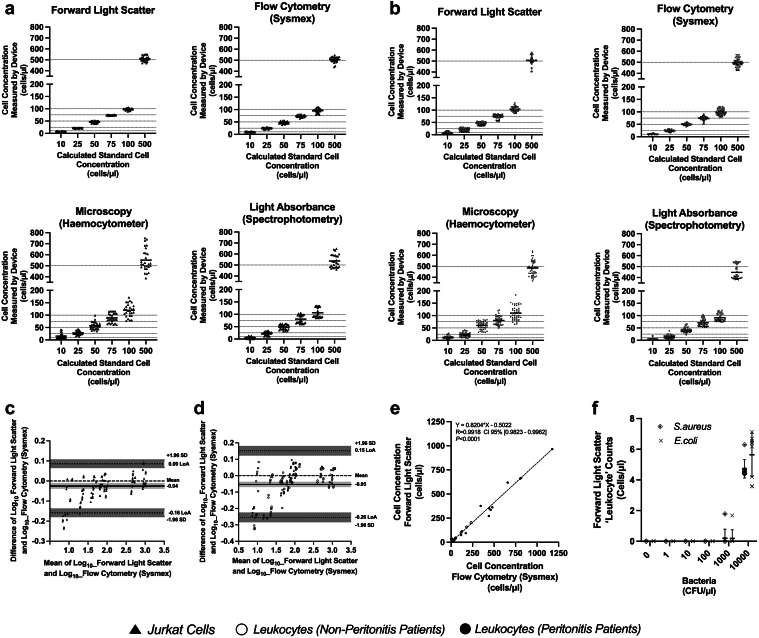
Accuracy, precision and bias of the forward light scatter device (QuickCheck) relative to flow cytometry (Sysmex), microscopy (haemocytometer) and light absorbance (spectrophotometric). (a) Accuracy and precision determined by measuring various standard dilutions of Jurkat cells, using the four cell counting methods. Calculated standard cell concentrations were obtained by diluting a single known Jurkat stock suspension. Measured cell concentration was the concentration reported by each device for each standard, *n* = 27 measurements per cell concentration. (b) Accuracy and precision of measurements of various dilutions of leukocytes from effluent from patients with or without peritonitis, using the four methods, and the same test as (a). Data shown is for 7 patient samples (2 with peritonitis and 5 without peritonitis) for QuickCheck and Sysmex *n* = 63 measurements per concentration, and 5 patient samples (2 with peritonitis and 3 without peritonitis) for haemocytometer and spectrophotometry *n* = 45 measurements per concentration. A summary of accuracy and precision values are provided in Table 2. (c) Bland-Altman analysis showing bias of log10 transformed measurements by QuickCheck relative to Sysmex for Jurkat cells (*n* = 126 comparisons) or (d) for leukocytes from effluent from patients with or without peritonitis (*n* = 147 comparisons). Solid horizontal line represents the mean bias, horizontal dashed line represents zero, dotted horizontal lines represent the 95% limits of agreement (LoA) ±1.96 SD of the mean bias. Shaded areas represent 95% CI of mean bias and upper LoA and lower LoA (e) Relationship between Sysmex and QuickCheck values for clinical samples from 13 non-peritonitis patients, 9 peritonitis patients and 7 patients with peritonitis on antibiotic therapy. Samples were measured in triplicate on each device and averaged. Dashed line shows a simple linear regression line of best fit. Correlation is shown using Pearson’s correlation co-efficient (R = 0.9918) and 95% CI [0.9823, 0.9962]. *P* < 0.0001 (two tailed, alpha 0.05). (f) QuickCheck apparent leukocyte count reported for samples only containing bacterial suspensions. Each bacterial suspension was prepared in triplicate (on 3 separate days) and measured in triplicate. Error bars represent SD and solid line represents the mean.

We constructed a forward light scatter instrument (QuickCheck) (Figure 3a–d), and measured the effect of various concentrations of Jurkat cells resuspended in PD patient effluent on scatter of 650–700 nm laser light, 1° to 12° from the incident axis. Scatter increased with cell number for all cell types and independently of cell type (Figure 3e). The performance of QuickCheck was compared with 3 alternative methods. Flow cytometry (Sysmex) and QuickCheck were most accurate for Jurkat cells and leukocytes from PD patients’ effluent, whereas hemocytometer and light absorbance (spectrophotometry) were least accurate. QuickCheck had the best precision (indicated by value nearest to 0%), followed by flow cytometry, light absorbance, and hemocytometer (Figure 4a and b, Table 2).

**Table 2:** Accuracy and precision of different cell counting methods

Cell counting method	Accuracy		Precision	
	Jurkat Cells	Patients’ Leukocytes	Jurkat Cells	Patients’ Leukocytes
Forward light scatter (QuickCheck)	96.8% ± 1.5%	94. 0% ± 3.4%	3.0% ± 0.4%	3.5% ± 1.3%
Flow cytometry (Sysmex)	97.5% ± 0.9%	95.7% ± 1.3%	5.8% ± 1.1%	6.2% ± 1.3%
Hemocytometer	86.0 % ± 6.8%	86.7% ± 4.2%	15.6% ± 1.6%	15.1% ± 2.9%
Light absorbance (Spectrophotometry)	83.4 % ± 9.7%	85.3% ± 5.6%	9.3% ± 7.2%	6.3% ± 2.9%

Data shown are for Jurkat cell cultures or PD patient’s leukocytes suspended at standard concentration in filtered PD effluent; over the cell concentration range from 50 to 1000 cells/μl, ±SD are shown. High accuracy is indicated by values closest to 100%, whereas high precision is indicated by values closest to 0% (see Supplementary Methods).

Bland-Altman analysis showed that 95% of results fall within the confidence intervals and that mean bias between QuickCheck and Sysmex is small, being −0.04 for Jurkat cells and −0.05 for leukocytes (Figure 4c and d). When QuickCheck counts were plotted against flow cytometry for unprocessed effluent samples directly from patients with or without peritonitis, we found a strong correlation (R = 0.9918)) between the methods (Figure 4e). This reliability between the 2 techniques may be summarized by an intraclass correlation coefficient, which is “excellent” for both Jurkat cells and leukocytes (0.98 [0.97–0.99 CI], *n* = 126 and 0.96 [0.93–0.97 CI], *n* = 147, respectively). We finally assessed the potential for QuickCheck leukocyte counts to be affected by high levels of bacteria, using various concentrations of *E* *coli* and *S* *aureus*. *S* *aureus* at 1 × 10^4^ CFU/μl (i.e., higher than what we detected from effluent from patients with peritonitis) resulted in apparent counts of 4.73 (SD: ±0.60) cells/μl and *E* *coli* with 5.64 (SD: ±1.42) cells/μl (Figure 4f).

## Discussion

The leukocyte concentrations we measured in patients on PD supports the relevance of the ISPD recommended threshold of 100 cells/μl, and this clearly distinguished almost all patients with and without peritonitis. Only 1 patient with peritonitis (2.7% of samples) had a total leukocyte count <100 cells/μl (80 cells/μl). Similarly, 3 patients without peritonitis had a leukocyte count >100 cells/μl (5.7% of samples); none had signs or symptoms, and were not subsequently diagnosed with peritonitis. In normal clinical practice, cytology would not have been performed, so this leukocyte elevation would presumably occur unnoticed. Our findings are consistent with a recent study proposing a slightly higher white blood cell count of 230 cells/μl as a PDRP threshold value and further research to refine the exact threshold value may be warranted.^20^ We detected diverse leukocytes, with neutrophils representing 12.4% (SD: ±12.5%), which is much lower than the >50% levels of polymorphonuclear granulocytes reported in patients with peritonitis.^10^ This is in line with previous reports of B-cells and activated T-cells in patients on continuous ambulatory peritoneal dialysis^21^^,^^22^ and leukocyte populations in nondialysis patients.^23^ These data support the use of total cell counts (in combination with signs and symptoms) to assist diagnosis in almost all patients.

The Sysmex data suggested bacterial colonization of effluent from patients without peritonitis, with this correlating with leukocyte concentration, and also confirmed that bacterial levels in patients with peritonitis, though higher, were limited. Presumably low level bacterial colonization of effluent does not invariably lead to peritonitis, and this is managed by leukocyte infiltration. Future studies using traditional culture techniques might confirm the presence and identity of these organisms.

WST-9-PP was the most suitable indicator for use with bacteria and mammalian cells, consistently providing a strong color change clearly visible to the eye. Selective color change for mammalian cells, vancomycin-sensitive, and vancomycin-insensitive bacteria was achieved for formulations 1, 2, and 3. Peritonitis diagnosis might be supported by the culture-positivity in formulations 1 and/or 2, color change indicating high leukocyte levels in formulation 3; and antibiotic selection could be modified according to vancomycin-sensitivity information.^24^ Nonetheless, the requirement for a 10-hour incubation at 37 °C, and the binary/nonquantitative leucocyte result are a limitation. However, conventional microbiological sensitivity testing is much slower, and in some clinics in remote locations, may not be immediately available. The leukocyte threshold occurs at around 100 to 300 cells/μl, representing approximately 33% (12/36) of peritonitis cases, and therefore there is a risk of false negatives for samples in this range. Bacterial color development typically occurs at 100 CFU/μl (in line with the mean bacterial count we found in peritonitis cases: 52 cells/μl). Bacterial levels may therefore be insufficient to produce color change with the current formulations for some peritonitis samples, meaning antibiotic sensitivity may not be available in all cases.

In contrast, the optical QuickCheck approach quantifies cell concentration within the range 10–1 × 10^4^ cells/μl. Bacterial samples as high as 1 × 10^4^ CFU/ml (greater than levels we detected in PD effluent, even in peritonitis samples) resulted in the instrument only detecting apparent leukocyte counts at levels below the concentration range reported on screen (and so the minimum result of “<10 cells/μl” is displayed). Although microbiological sensitivity information is not provided, rapid and accurate leukocyte counting at POC would enable monitoring of patients during antibiotic therapy, and antibiotics to be changed and/or aid decision making with regard to PD catheter removal^10^ if leukocyte count does not decrease.

Although our study did not aim to test the diagnostic sensitivity and specificity of the ISPD diagnostic guidelines, we did measure the relative accuracy, precision, and bias of the novel POC light scatter instrument. QuickCheck showed similar accuracy to and better precision than the laboratory flow cytometer (Sysmex UF-5000), an instrument used in 4 of 8 hospitals in the study, for total leukocyte counting. The linearity of Sysmex counts plotted against QuickCheck counts, and minimal bias apparent from the Bland-Altman analysis, further confirms the equivalence of these 2 methods. Hemocytometer counting and spectrophotometric assessment using a Biochrom Libra S12 laboratory spectrophotometer, both resulted in lower accuracy and precision. Hemocytometer counting alone is used in 4 of 8 hospitals in the study, and its relative inaccuracy was therefore surprising, perhaps reflecting its inherent subjectivity. Absorbance directly quantifies cloudiness and would be affected by any substance absorbing light, including bacteria, fibrin, or chylomicrons, potentially explaining its inaccuracy.

The use of QuickCheck with patients presenting with suspected peritonitis, could reduce time to diagnosis and administration of antibiotics. Median time from first clinician contact to administration of antibiotics was 3.4 hours for patients with PDRP who subsequently lost their catheter or died.^11^ An immediate leukocyte count would (i) enable patients with suspected peritonitis, where diagnosis is not supported by QuickCheck, to be discharged promptly without the need to wait in clinic and (ii) ensure as short a time as possible from suspected diagnosis to antibiotic administration, thereby diminishing the increased risk of technique failure and mortality associated with treatment delay.^11^ Use by assisted care or community nurses to follow up peritonitis patients 3 to 5 days after diagnosis at home may prevent a second hospital visit, confirm patients’ recovery, or highlight possible refractory peritonitis.^10^ As such, it is important that the instrument can accurately quantity visible higher levels of leukocytes, to enable tracking of these levels after antibiotic therapy is begun.

In addition, many patients have visual difficulties, making “bare eye inspection” of PD effluent unreliable, supporting the utility of a near-patient rapid test. Indeed, QuickCheck might be used directly by patients at home, especially those at high risk of peritonitis due to a history of recurrent peritonitis, touch contamination, frailty or demographic, or patients living at long distance from the clinic or where transport is limited or where visual impairment affects the ability to detect a change in the appearance of the effluent. More frequent home visits and support for patients increases peritonitis-free survival^25^; access to QuickCheck may have comparable effects, especially for the higher risk groups.

In conclusion, we have developed and tested 2 complementary methods for supporting POC peritonitis identification and management in patients with PD. The first provides some antibiotic sensitivity information, but requires 10 hours incubation, whereas the second optical approach (QuickCheck), provides instant accurate total leukocyte count and may have greater clinical utility to assist in diagnosis.

## Disclosure

NG-B, SMK, MGB, DK, DH, AYG, ASM, TW, and RG are employees of MicroBioSensor Ltd.; PF, WK, NG, CGK, and CBD are directors of or consultants to MicroBioSensor Ltd.; and JQ and AV have received research support from MicroBioSensor Ltd. All the other authors declared no competing interests.

## Supplementary Material

Supplementary file (PDF)

**Supple** **mentary Methods.**

**Figure S1.** Comparison of the visual appearance of Jurkat cells at a range of cell concentrations resuspended in patient PD effluent. Mock effluent was in CAPD drain bags which were placed over text (as patients are directed in the ISPD guidelines). Images show top panel left to right, 0, 10, and 50 cells/μl. Bottom panel left to right shows 100, 200, and 500 cells/μl. Images shown are representative of typical results (all experiments were carried out in triplicate). The appearance of images have not been altered or modified from their original format at the time of capture.

**Figure S2.** Development of 3 selective tetrazolium formulations for visual identification of above threshold levels of bacteria, vancomycin-insensitive bacteria, or total leukocytes. (A) Comparison of the visual intensity of five tetrazolium compounds after challenge with 0 to 10^6^ CFU/ μl gram-positive or gram-negative bacteria, or 0 to 10^3^ HL60 cells/ μl, after 10 h incubation. (B) Effect of various concentrations of the cytotoxin digitonin on the ability of HL60 or six bacterial strains to reduce WST9 -PP and produce a visible colour change. (C) Selectivity of the colour change for a WST-9-PP formulation containing 0.005% digitonin, tested using mixtures of various concentrations of HL60 cells and *E**coli* or *S**aureus*. (D) Extent of inhibition of colour development for WST9-PP by inclusion of up to three antibiotics (vancomycin (V), ciprofloxacin (C) and / or meropenem (M) tested against 13 bacterial strains and HL60 cells (control). (E) Response of WST9 -PP formulation containing vancomycin, ciprofloxacin and meropenem to challenge with mixtures of various concentrations of HL60 cells and *E**coli* or *S**aureus*. (F) Response of WST9-PP formulations 1 and 2 challenged with 100 CFU/ μl of the vancomycin-sensitive gram-positive organisms *S**aureus* and *S**epidermidis* or the gram-negative organisms *E**coli* and *P**aeruginosa*. Images are representative of typical results (all experiments were carried out in triplicate). The appearance of images have not been altered or modified from their original format at the time of capture.

**Table S1.** Antibodies and associated metal tags used for mass cytometric analysis (Fluidigm).

**Table S2.** Vancomycin minimum inhibitory concentration (MIC) for various vancomycin-sensitive and vancomycin-insensitive bacteria.

**Table S3.** Chemical constituents of 3 selective formulations, showing weights of each per pharmaceutical capsule (mg/capsule).

**Supplementary Reference**

**SAMPLE Checklist**
