# Supplementary material for: Novel Colorimetric and Light Scatter Methods to Identify and Manage Peritoneal Dialysis-Associated Peritonitis at the Point-of-Care
Source: Kidney Int Rep. 2023 Dec 30;9(3):589–600. doi: 10.1016/j.ekir.2023.12.021 (PMC10927466; doi:10.1016/j.ekir.2023.12.021)
Supplement: Supplementary file (PDF) [file mmc1.pdf]

Supplementary Methods

Mass Cytometry Sample Processing

We isolated cells by centrifugation (300 g, 10 min) from 50ml of effluent from nine PD patients without peritonitis. After two PBS washes (resuspension and further centrifugation at 300g, 5min), 100μl cisplatin solution was added (Cell-ID Intercalator-cisplatin, Fluidigm) and samples incubated (2min). Next 500μl of PBS containing 10% BSA (Sigma Aldrich) (cell staining buffer) was added and samples subjected to centrifugation (300g, 5min), followed by resuspension in 100μl cell surface antibody cocktail (Supplementary Table S1), incubation (20min), and dilution with 1ml PBS. After centrifugation (300g, 5min) 100μl permeabilization/ fixation buffer (BD Biosciences) was added and samples incubated (20min). After further centrifugation (300g, 10min) washing in 1ml perm/wash buffer (BD Bioscience) and further centrifugation (300g, 10min) 100μl Ir solution (Cell-ID Intercalator-Ir, Fluidigm) was added overnight at 4°C, before centrifugation (300g, 10min) and resuspension in FBS containing 10% DMSO (Sigma Aldrich). Samples were stored (-80°C) prior to analysis using a Helios mass cytometer (Fluidigm).

Tetrazolium colour change visibility assay

Experiments with bacteria (*S.aureus* 6538P and *E.coli* 8739) used Wilkins Chalgren Broth (WCB) (Oxoid Ltd, Hampshire, UK) at the concentration recommended by the manufacturer. HL-60 cell experiments were carried out in pooled effluent, filtered using a 0.22μm filter and buffered by the addition of 25mM HEPES (Sigma-Aldrich). We tested a number of tetrazolium compounds, all of which are used commercially in cell viability assays. These compounds are metabolised by mammalian, bacterial and fungal cells, resulting in the production of a formazan product, which has a markedly different colour from the non-metabolised tetrazolium. Specifically, thiazolyl blue tetrazolium bromide (MTT) (final concentration 0.1mg/ml), nitro blue tetrazolium (NBT) (final concentration 0.2mg/ml), tetrazolium violet (TV) (final concentration 0.2mg/ml), blue tetrazolium chloride (BTC) (Sigma) (final concentration 0.3mg/ml) or water-soluble tetrazolium 9 (WST-9) (Concept Life Sciences, High Peak, UK) (final concentration 0.18mg/ml) were tested. For WST-9, polyvinylpyrrolidone (PVP) (Sigma-Aldrich) and the electron mediator 1-methoxy-5-methylphenazinium methyl sulfate (mPMS) (Glentham, Wiltshire, UK) were also added to the test medium (0.8mg/ml and 8.4μg/ml final concentrations respectively), with this

mixture being referred to as WST-9-PP. In preliminary experiments mPMS and PVP were found to enhance WST-9 colour development, by 10h**;** there was no impact on colour development for the other tetrazolium compounds, and so these were only included for WST-9. Various concentrations of bacterial suspension (in the range 1-10^6 CFU/µl) or HL60 cell suspensions (in the range 10-10^4 cells/µl) were prepared containing the tetrazolium salts, in 96-well plates (200µl/well) for bacteria or 48-well plates (500µl/well) for cells. These were then incubated for 10h at 37°C without agitation, before visual examination for colour development, and imaging of the plates (Brinno timelapse camera, Taiwan).

Development of selective indictor media for the three formulation IVD

We intended formulations 1 and 2 to contain digitonin, to prevent growth of any mammalian cells leading to colour change from the WST-9-PP present. Formulation 2 should additionally contain a discriminatory concentration of vancomycin, to prevent growth of vancomycin-sensitive organisms. Formulation 3 contained the antibiotics meropenem, ciprofloxacin and/ or vancomycin, to enable only cellular growth to occur.

Determining digitonin levels required to prevent mammalian cell growth in the microbiologically sensitive formulations

The microbial base indicator media for the two formulations sensitive to microbial growth comprised WCB (11.6mg/ml), WST-9 (0.38mg/ml), mPMS (8.4µg/ml), PVP (0.8mg/ml). This was supplemented with digitonin (Biosynth Carbosynth, Compton, UK) added at various concentrations from 0.0006% to 0.01% (w/v), to establish levels able to inhibit mammalian cell growth, without inhibiting bacterial growth. Digitonin was added by preparing a 100x concentrated stock and adding this to obtain the desired 2x concentration in a final volume of 100 µl, before twofold dilution of digitonin by addition of 100 µl of HL60 cell suspensions at various concentrations (5×10^3, 1×10^4 and 2×10^4 cells/µl). Plates were incubated for 10 h at 37°C and examined for colour development. Additionally, we tested the effect of 0.005% (w/v) digitonin on the colour change caused by six representative bacterial strains: *S. aureus* (ATCC 6538P), *S. epidermidis* (ATCC 35984), *E. faecalis* (ATCC 51299), *K. pneumoniae* (ATCC 8044), and *P. aeruginosa* (ATCC 9027) after inoculation of the same media with bacteria at 1×10^2 CFU/µl, incubated at 37°C for 10h.

Determination of MIC for vancomycin, for use in inhibiting vancomycin-sensitive organisms

Decreasing concentrations of vancomycin were prepared in serial twofold dilutions in 96-well plates using microbial base indicator media in 100µl; growth controls without antibiotic were also included. Next, 100µl of bacterial suspension ($2x10^3$CFU/µl) also in microbial base indicator media, was added (resulting in final vancomycin concentrations after this further twofold dilution of 0.5-64µg/ml). After 10h incubation at 37°C, plates were examined for colour development indicating bacterial growth. The MIC was defined as the lowest concentration that inhibited the growth of all Gram-positive organisms. Finally, the inhibitory effect of the proposed formulation 2 media (microbial base medium containing 16µg/µl vancomycin) relative to formulation 1 media (microbial base media without vancomycin) was tested by inoculating 16ml of each media in flexible plastic chambers with four organisms (*S. aureus* (ATCC 6538P), *S. epidermidis* (ATCC 14490), *E. coli* (ATCC 8739) and *P. aeruginosa* (ATCC 9027) at 1×10^3 CFU/µl, and incubating at 37°C for 10h before observation.

Determination of antibiotics needed to completely inhibit bacterial growth in the leukocyte formulation without affecting cell growth (formulation 3).

Cell base indicator media comprised WST-9 (0.38mg/ml), mPMS (16.8µg/ml), PVP (2.2mg/ml), WCB (3.46mg/ml) resuspended in filtered PD effluent. Three antibiotics were tested at the following (high) concentrations: 16µg/ml meropenem (Glentham, Wiltshire, UK), 16µg/ml ciprofloxacin (ARCOS Organics, Geel, Belgium) and 16µg/ml vancomycin (Phion, Dorset, UK), individually or in combination. Thirteen bacterial strains at 1×10^4CFU/µl were introduced in pooled filtered effluent (0.22µm) 96-well plates respectively and incubated for 10h at 37°C without agitation. Plates were visually examined for colour development images were acquired using a lightbox and time-lapse colour camera. Additionally, HL60 cells in 48-well plates (500µl/well) were grown in cell base indicator media containing all three antibiotics at a range of cell concentrations (0, 50, 100, 300, 500, 1×10^3cells/µl).

Confirmation of microbial and cell selectivity of indicator media for use in IVD.

The final composition of indicator formulations 1, 2 and 3 are shown in Supplementary Table S2. Each formulation was filled with 16ml of relevant media plus bacteria and/or HL-60 cells. The overall selectivity of formulation 1 for bacteria was confirmed by testing this using

various concentrations of *E coli* ATCC 8739 or *S aureus* (ATCC 6538) (10–1 x 10^4CFU/µl) mixed with various HL60 concentrations (50–1 x 10^3cells/µl), in 96-well plates. Colour change was monitored after 10h incubation at 37°C. The overall selectivity of formulation 3 for mammalian cells was confirmed by testing this formulation using various concentrations of HL60 cells (100–1 x 10^4 cells/µl), mixed with either *E coli* ATCC 8739 or *S aureus* (ATCC 6538) (1–1 x 10^4 CFU/µl) in 96-well plates. Again, colour change was monitored after 10h incubation at 37°C.

Measuring colour change in full size cassettes

To visualise colour change within the cassettes, indicator formulations were inoculated with *S. aureus* (ATCC 6538P), *K pneumoniae* (ATCC 8044) (both at 1 x 10^2CFU/µl) or HL60 cells (at 5 x 10^2cells/µl). Falcon tubes, and 16ml of each transferred using a sterile syringe into a bespoke polyvinylchloride (PVC) chamber (with a translucent front, and white plastic rear surface) housed in a cassette. These were sealed and incubated for 10h at 37°C, before visual assessment of colour development.

Appearance of peritoneal dialysis effluent in drain bags

Jurkat cells were prepared at final cell concentrations of 0, 10, 50, 100, 200, 500 cells/µl in 0.22µm filtered effluent in 1mL volume for each cell concentration. 2L continuous ambulatory PD drain bags (Baxter Healthcare Ltd, Norfolk, UK) were filled with each cell suspension and the clear window placed on text to assess visibility through sample.

Accuracy, precision and bias for four counting methods

Experiments with Jurkat cells were replicated in triplicate, with three samples per experiment for each cell concentration, with 3ml of each sample then added into polystyrene macrocuvettes (FisherBrand, Leicestershire, UK), and each read in triplicate using the QuickCheck device, and by spectrophotometry at 600nm (Libra S12 spectrophotometer, Biochrom Ltd, Cambridge, UK). Clinically derived leukocytes were isolated from 7 peritoneal dialysis patients (2 with peritonitis and 5 without peritonitis), with three samples per patient for each cell concentration, read in triplicate as described above. Cell numbers were extrapolated from standard curves of known cell concentrations generated on the spectrophotometer. Manual cell counts were obtained using a Neubauer haemocytometer (Sigma-Aldrich) using 10µl of each sample. Sysmex UF-5000 counts were performed using Body Fluid mode, calibrated using UF control beads using 600µl of each sample. For each approach we determined the average measured value for each of the test

dilutions. The average accuracy and precision were determined for each cell concentration and results averaged across the range of 50-1000 cells/µl. The accuracy of each counting method was defined as (100%)-(%Error), with %Error defined as |(Measured value-True value)| / (True value x 100%). The precision of each was determined by calculating its co-efficient of variation (%CV) in achieving each target cell concentration. This was carried out for all reads at each cell concentration, and defined as SD / mean | x 100%.

A further comparison was made between the QuickCheck and Sysmex UF-5000 devices, using seven test cell concentrations (10, 25, 50, 75, 100, 500 and 1 x 10^3 cells/µl) measured in triplicate on each device (Six independent experiments performed for Jurkats cells n=126 comparisons and seven for Clinically derived leukocytes n=147 comparisons). Data was Log_{10} transformed and bias was calculated using Bland-Altman analysis and 95% confidence intervals determined using MedCalc® Statistical Software version 22.009 (MedCalc Software Ltd, Ostend, Belgium; https://www.medcalc.org; 2023).

Statistical analysis: different sample sizes

The sample sizes in Fig. 1a-c are very different among the treatments. To check that this had no effect on the significance of our findings we randomly resampled our data with replacement (bootstrap resampling), to make all sample sizes equal to the smallest in the comparison. While the overall reduction in sample size reduces the power of the tests, meaning the bootstrap P values are generally higher than the main analysis, the significance or otherwise of the tests remains as shown in Fig. 1. Median bootstrap P values with (interquartile ranges): Fig. 1a P = 1.7 x 10^{-12} (7.4 x 10^{-13} – 4.7 x 10^{-12}) ; Fig. 1b P = 0.31 (0.10 – 0.63) ; Fig. 1c P(non-Peritonitis versus Peritonitis with Antibiotic therapy) 0.013 (0.0035 – 0.044), P(non-Peritonitis versus suspected Peritonitis) = 0.00089 (0.00018 – 0.0044), P(Peritonitis with Antibiotic therapy versus suspected Peritonitis) = 0.32 (0.13 – 0.61).

Statistical analysis: intraclass correlation coefficient (ICC).

We calculated the ICC [s1] for readings taken by QuickCheck and Sysmex from mixed effects models containing fixed effects of Cell number, machine and their interaction and a random effect of sample, separately for Jurkat cells and leukocytes (there was no discernible variance attributable to different dilution series tested for either set of cells). The ICC was taken as the variance among samples as a proportion of the total variance, with confidence intervals calculated by profile likelihood. The model was fitted and assessed using the lme4 package v1.1 [s2] in R v4.3 [s3].

Supplementary Figures

Supplementary Figure S1. Comparison of the visual appearance of Jurkat cells at a range of cell concentrations resuspended in patient PD effluent. Mock effluent was in CAPD drain bags which were placed over text (as patients are directed in the ISPD guidelines). Images show top panel left to right, 0, 10 and 50 cells/µl. Bottom panel left to right shows 100, 200 and 500 cells/µl. Images shown are representative of typical results (all experiments were carried out in triplicate). The appearance of images have not been altered or modified from their original format at the time of capture.

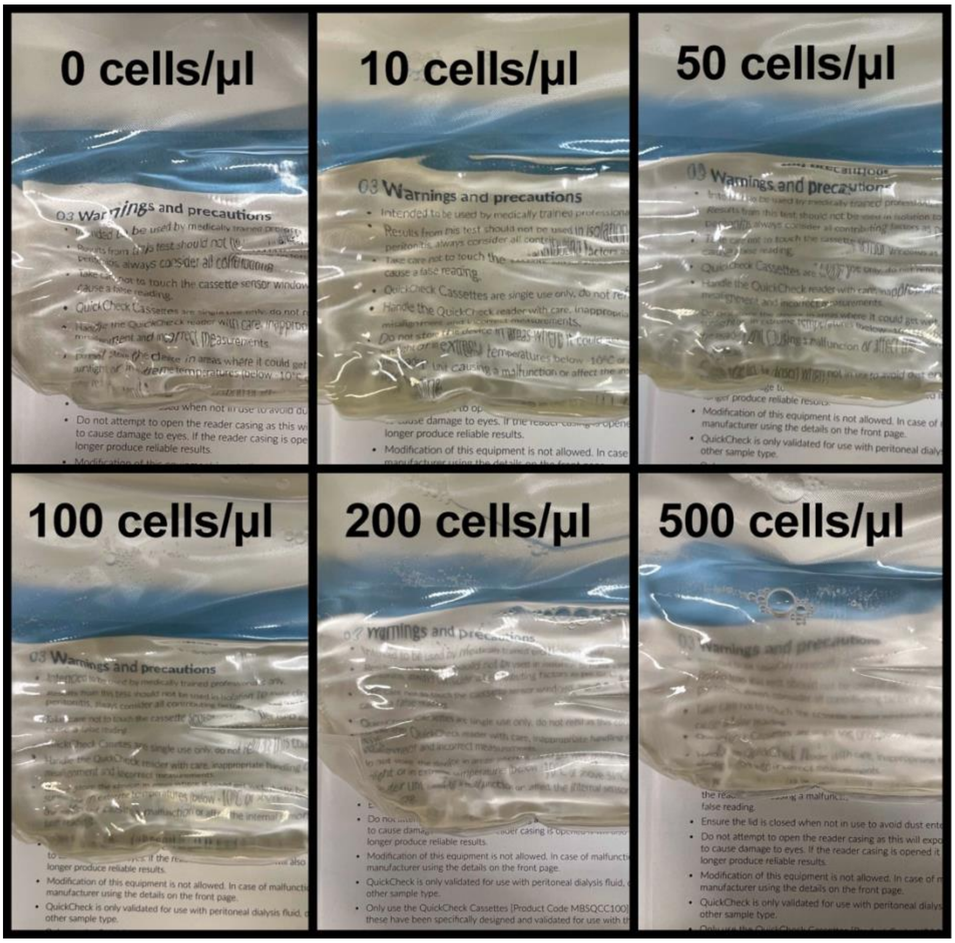

Supplementary Figure S2. Development of three selective tetrazolium formulations for visual identification of above threshold levels of bacteria, vancomycin-insensitive bacteria or total leukocytes. (a) Comparison of the visual intensity of five tetrazolium compounds after challenge with 0 to 10^6 CFU/µl gram-positive or gram-negative bacteria, or 0 to 10^3 HL60 cells/µl, after 10 h incubation. (b) Effect of various concentrations of the cytotoxin

digitonin on the ability of HL-60 or six bacterial strains to reduce WST-9-PP and produce a visible colour change. (c) Selectivity of the colour change for a WST-9-PP formulation containing 0.005% digitonin, tested using mixtures of various concentrations of HL60 cells and *E. coli* or *S. aureus*. (d) Extent of inhibition of colour development for WST-9-PP by inclusion of up to three antibiotics (vancomycin (V), ciprofloxacin (C) and / or meropenem (M) tested against 13 bacterial strains and HL60 cells (control). (e) Response of WST-9-PP formulation containing vancomycin, ciprofloxacin and meropenem to challenge with mixtures of various concentrations of HL60 cells and *E. coli* or *S. aureus*. (f) Response of WST-9-PP formulations 1 and 2 challenged with 100 CFU/µl of the vancomycin-sensitive gram-positive organisms *S. aureus* and *S. epidermidis* or the gram-negative organisms *E. coli* and *P. aeruginosa.* Images are representative of typical results (all experiments were carried out in triplicate). The appearance of images have not been altered or modified from their original format at the time of capture.

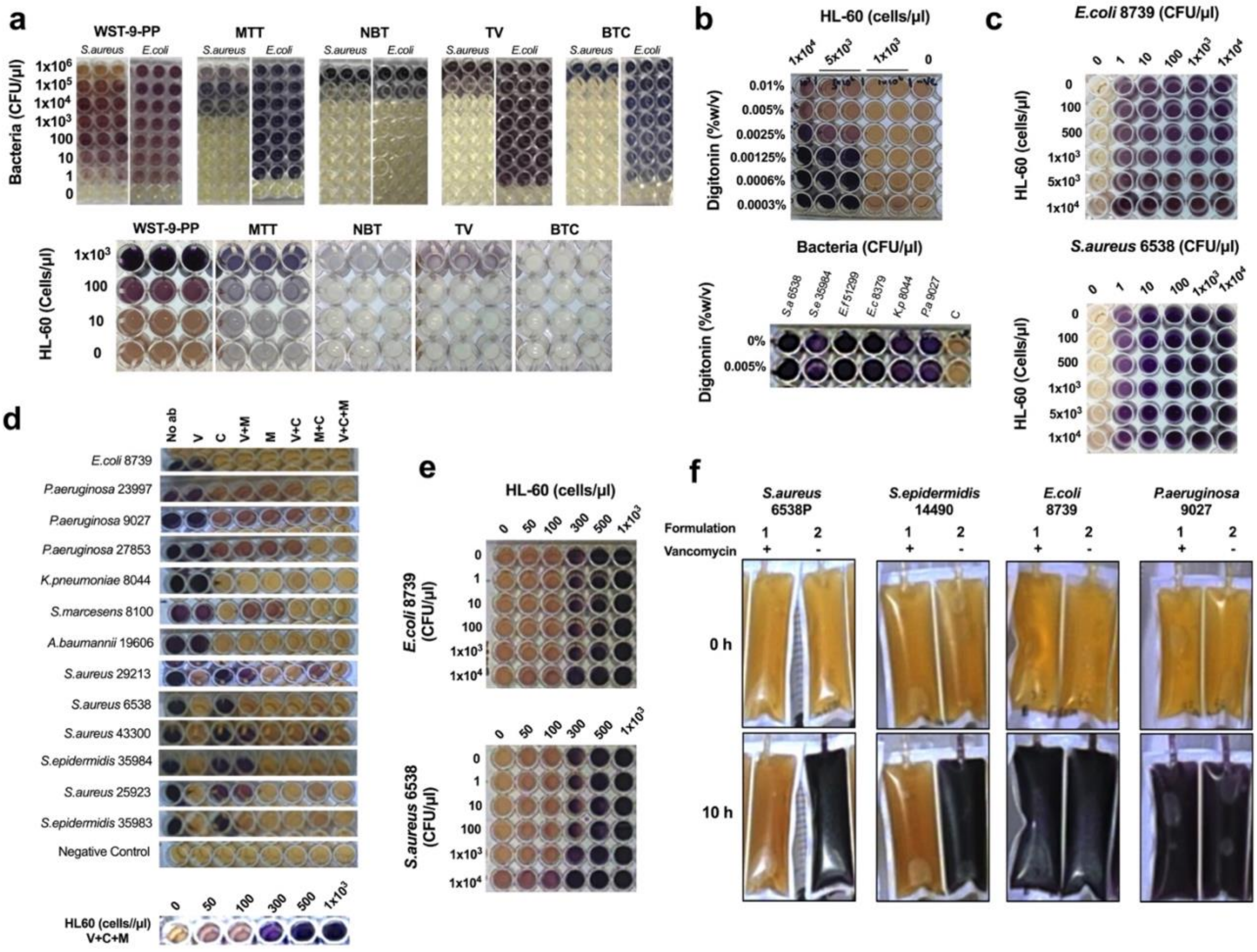

Supplementary Table S1. Antibodies and associated metal tags used for mass cytometric analysis (Fluidigm).

Marker	Metal Tag	Isotope	Cellular target	Fluidigm catalogue number
CD45	Y	89	Immune cell	3089003B
CD3	Er	170	T cell	3170001B
CD4	Nd	145	T helper cell	3145001B
CD8	Nd	146	Cytotoxic T cell	3146001B
CD19	Nd	142	B cell	3142001B
CD33	Gd	158	Myeloid cell	3158001B
CD14	Nd	148	Monocyte	3148010B
CD16	Ho	165	Monocyte / macrophage	3165001B
CD56	Sm	149	Natural killer cell	3149021B
CD66b	Dy	162	Neutrophil	3162023B
HLA-DR	Yb	173	Antigen Presenting cell	3173005B
CD11b	Er	167	Dendritic cell	3167011B
CD303	Eu	153	Plasmacytoid dendritic cell	3153007B
EpCam	Pr	141	Epithelial cell	3141006B

Supplementary Table S2. Chemical constituents of three selective formulations, showing weights of each per pharmaceutical capsule (mg/capsule).

	**Formulation 1**		**Formulation 2**		**Formulation 3**	
	Capsule A	*Capsule B*	*Capsule A*	*Capsule B*	*Capsule A*	*Capsule B*
WST-9	6.05		6.05		6.05	
mPMS	0.13		0.13		0.27	
Digitonin	0.80		0.80			
Mannitol	116.2	37	116.2	37	26	6.05
Wilkins Chalgren Media		185		185		55.4

PVP	12.8		12.8		35.5
Vancomycin		0.256		0.128	
MES				78.1	
Meropenem				0.256	
Ciprofloxacin				0.128	
Sodium Bicarbonate					3.00

Supplementary Table S3. Vancomycin minimum inhibitory concentration (MIC) for various vancomycin-sensitive and vancomycin-insensitive (*) bacteria.

**Bacteria / Strain (ATCC)**	**Vancomycin MIC (μg/ml)**
S. aureus (6538)	0.5
S. aureus (25923)	1
S. aureus (29213)	1
MRSA (43300)	1
S. epidermidis (12228)	1
S. epidermidis (35983)	1
S. epidermidis (14990)	1
*E. coli (3987) **	>64
*P. aeruginosa (9027) **	>64
*P. aeruginosa (23997) **	>64
*P. aeruginosa (23853) **	>64
*K. pneumoniae (8044) **	>64
*S. marcescens (8100) **	>64
*A. baumanii (19606) **	>64

Supplementary References

S1.Koo TK, Li MY. A Guideline of Selecting and Reporting Intraclass Correlation Coefficients for Reliability Research. *J Chiropr Med*. 2016;15(2):155-163. doi:10.1016/j.jcm.2016.02.012.

S2. Bates D, Mächler M, Bolker B, Walker S. Fitting Linear Mixed-Effects Models Using lme4. *J Stat Softw*. 2015;67(1). doi:10.18637/jss.v067.i01

S3. R core team. A Language and Environment for Statistical Computing. R foundation for statistical computing, Vienna. https://www.R-project.org/ Date accessed: December 4, 2023.

SAMPL Checklist

Statistics reporting

For all statistical analyses, we can confirm that the following items are present in the manuscript according to the SAMPL Guideline.
(https://www.equator-network.org/wp-content/uploads/2013/07/SAMPL-Guidelines-6-27-13.pdf Lang T, Altman D. Reporting Basic Statistical Analyses and Methods in the Published Literature: The SAMPL Guidelines for Biomedical Journals)

Location	**Preliminary Analyses: relevant for Fig 1 and 4**
Page 10	Identify any statistical procedures used to modify raw data before analysis. Examples include mathematically transforming continuous measurements to make distributions closer to the normal distribution, creating ratios or other derived variables, and collapsing continuous data into categorical data or combining categories
	**Primary Analyses: relevant for Fig 1 and 4**
Page 10	Describe the purpose of the analysis.
Page 10	Identify the variables used in the analysis and summarize each with descriptive statistics.
Page 10	When possible, identify the smallest difference considered to be clinically important.
Methods, Supplementary and Fig captions	Describe fully the main methods for analyzing the primary objectives of the study.
Page 10 and in Fig 1 and 4 figure captions	Make clear which method was used for each analysis, rather than just listing in one place all the statistical methods used.
Pages 10, 11 and Fig 1 and 4 caption	Verify that that data conformed to the assumptions of the test used to analyze them. In particular, specify that 1) skewed data were analyzed with non-parametric tests, 2) paired data were analyzed with paired tests, and 3) the underlying relationship analyzed with linear regression models was linear.
Page 10 and Fig 1 and 4 caption	Indicate whether and how any allowance or adjustments were made for multiple comparisons (performing multiple hypothesis tests on the same data).
N/A	If relevant, report how any outlying data were treated in the analysis.
Page 10 and Fig 1 and 4 caption	Say whether tests were one- or two-tailed and justify the use of one-tailed tests.
Page 10	Report the alpha level (e.g., 0.05) that defines statistical significance.
Page 10 and Fig 1 and 4 caption and Supplementary methods	Name the statistical package or program used in the analysis.
	**Reporting numbers and descriptive statistics: relevant for Fig 1b, 1f, 3e and 4f**
Page 10, 11, 14, and Fig 1 caption	Report numbers—especially measurements—with an appropriate degree of precision. For ease of comprehension and simplicity, round to a reasonable extent.

Page 11 and Fig 1 and 4 caption and supplementary methods	Report total sample and group sizes for each analysis.
Page 11	Summarize data that are approximately normally distributed with means and standard deviations (SD).
Page 11 and Fig 4 caption	Summarize data that are not normally distributed with medians and interpercentile ranges, ranges, or both. Report the upper and lower boundaries of interpercentile ranges and the minimum and maximum values of ranges, not just the size of the range.
Page 11 and Fig 1, 3 and 4 caption	Do NOT use the standard error of the mean (SE) to indicate the variability of a data set. Use standard deviations, inter-percentile ranges, or ranges instead.
Page 18	Display data in tables or figures. Tables present exact values, and figures provide an overall assessment of the data.
	**Reporting risk, rates and ratios: relevant for Fig 4a and 4b**
N/A	Identify the type of rate (e.g., incidence rates; survival rates), ratio (e.g., odds ratios; hazards ratios), or risk (e.g., absolute risks; relative risk differences), being reported.
Page 11	Identify the quantities represented in the numerator and denominator.
N/A	Identify the time period over with each rate applies.
N/A	Identify any unit of population (that is, the unit multiplier: e.g., x 100; x 10,000) associated with the rate.
Page 13 and Fig 1 and 4 caption	Consider reporting a measure of precision (a confidence interval) for estimated risks, rates, and ratios.
	**Reporting correlation analyses: relevant for Fig 1 and Fig 4c, 4d, 4e**
Page 10	Describe the purpose of the analysis.
Page 10	Summarize each variable with the appropriate descriptive statistics.
Page 13 and Fig 1 and 4 caption	Identify the correlation coefficient used in the analysis (e.g., Pearson, Spearman).
Page 15	Confirm that the assumptions of the analysis were met.
Page 10 and Fig 1 and 4 caption	Report the alpha level (e.g., 0.05) that indicates whether the correlation coefficient is statistically significant.
Page 14 and Fig 4 caption	Report the value of the correlation coefficient. Do not describe correlation as low, moderate, or high unless the ranges for these categories have been defined. Even then, consider the wisdom of using these categories given their biological implications or realities.
Page 10 and Fig 1 caption	For primary comparisons, report the (95%) confidence interval for the correlation coefficient, whether or not it is statistically significant.
Page 10 and Fig 1 and 4 caption	For primary comparisons, consider reporting the results as a scatter plot. The sample size, correlation coefficient (with its confidence interval), and P value can be included in the data field.
Page 10 and Fig 1 caption	Name the statistical package or program used in the analysis.

Image selection

We can confirm that the following items have been considered in selecting and presenting/reporting of images in the manuscript as referenced on the Equator network :

☐ ☐

https://www.equator-network.org/reporting-guidelines/selection-and-presentation-of-imaging-figures-in-the-medical-literature/

Siontis GC, Patsopoulos NA, Vlahos AP, Ioannidis JP. Selection and presentation of imaging figures in the medical literature. PLoS One. 2010;5(5).

Location	**Relevant for Figure 2 and Supplementary Figures S1 and S2**
Fig 2 and Fig S1 and S2 caption	*Selection process of the published image: Clarify if the intention to present an average case, an extreme case, or a selected case. If an extreme or a selected case, then clarify on what aspect.*
Fig 2 and Fig S1 and S2 caption	*Contrast specification: Clarify whether contrast is used in the study in all or some images, and if so provide sufficient detail to allow understanding whether contrast has been used in each specific image.*
